# Single-walled Carbon Nanotubes Wrapped with Charged Polysaccharides Enhance Extracellular Electron Transfer

**DOI:** 10.1021/acsabm.4c00749

**Published:** 2024-07-30

**Authors:** Tomohiro Shiraki, Yoshiaki Niidome, Arghyamalya Roy, Magnus Berggren, Daniel T. Simon, Eleni Stavrinidou, Gábor Méhes

**Affiliations:** † Department of Applied Chemistry, Kyushu University, 744 Motooka, Nishi-ku, Fukuoka 819-0395, Japan; ‡ International Institute for Carbon-Neutral Energy Research (WPI-I2CNER), Kyushu University, 744 Motooka, Nishi-ku, Fukuoka 819-0395, Japan; § Laboratory of Organic Electronics, Department of Science and Technology, Linköping University, Bredgatan 33, Norrköping 601 74, Sweden; ∥ Wallenberg Wood Science Center, Department of Science and Technology, Linköping University, Bredgatan 33, Norrköping 601 74, Sweden; ⊥ Graduate School of Information, Production and Systems, Waseda University, Hibikino 2-7, Wakamatsu, Kitakyushu 808-0135, Japan

**Keywords:** carbon nanotubes, extracellular electron transfer, *Shewanella oneidensis*, microbial electrochemical system, biological interaction

## Abstract

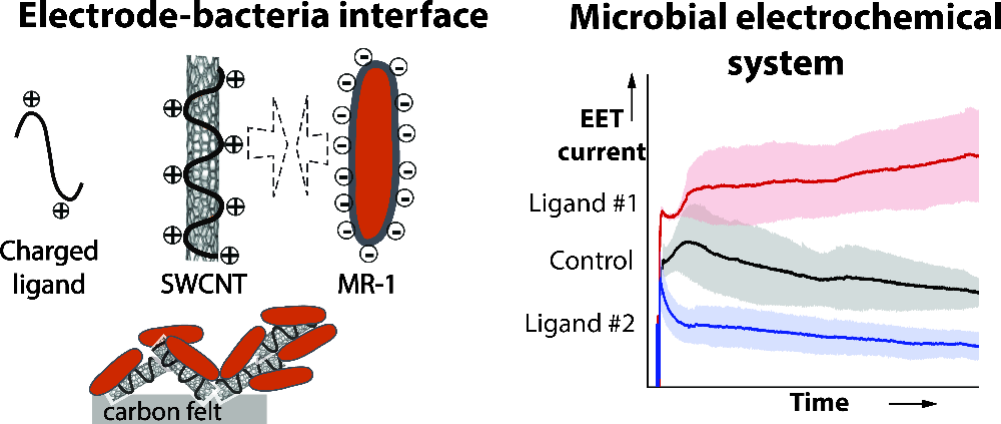

Microbial electrochemical systems (MESs) rely on the microbes’ ability to transfer charges from their anaerobic respiratory processes to electrodes through extracellular electron transfer (EET). To increase the generally low output signal in devices, advanced bioelectrical interfaces tend to augment this problem by attaching conducting nanoparticles, such as positively charged multiwalled carbon nanotubes (CNTs), to the base carbon electrode to electrostatically attract the negatively charged bacterial cell membrane. On the other hand, some reports point to the importance of the magnitude of the surface charge of functionalized single-walled CNTs (SWCNTs) as well as the size of functional groups for interaction with the cell membrane, rather than their polarity. To shed light on these phenomena, in this study, we prepared and characterized well-solubilized aqueous dispersions of SWCNTs functionalized by either positively or negatively charged cellulose-derivative polymers, as well as with positively charged or neutral small molecular surfactants, and tested the electrochemical performance of *Shewanella oneidensis* MR-1 in MESs in the presence of these functionalized SWCNTs. By simple injection into the MESs, the positively charged polymeric SWCNTs attached to the base carbon felt (CF) electrode, and as fluorescence microscopy revealed, allowed bacteria to attach to these structures. As a result, EET currents continuously increased over several days of monitoring, without bacterial growth in the electrolyte. Negatively charged polymeric SWCNTs also resulted in continuously increasing EET currents and a large number of bacteria on CF, although SWCNTs did not attach to CF. In contrast, SWCNTs functionalized by small-sized surfactants led to a decrease in both currents and the amount of bacteria in the solution, presumably due to the detachment of surfactants from SWCNTs and their detrimental interaction with cells. We expect our results will help researchers in designing materials for smart bioelectrical interfaces for low-scale microbial energy harvesting, sensing, and energy conversion applications.

## 1 Introduction

The Gram-negative bacterium *Shewanella oneidensis* MR-1 (*S. oneidensis* MR-1) is a major subject of studies that investigate, engineer, or make use of the coupling of metabolic energy generation to respiratory electron transfer to the external environment. This electron transfer is termed extracellular respiration or extracellular electron transfer (EET). *S. oneidensis* MR-1 is able to perform direct chemical-to-electrical signal/energy conversion when interfacing with charge collectors (electrodes) of electrical devices, such as microbial electrochemical systems (MESs, used for sensing analytes such as arabinose,^1^ arsenic,^2^ and lactate^3^), as well as for electrosynthesis^4^ including radical polymerization.^5^ Other devices similarly relying on EET as their driving mechanism are microbial fuel cells (MFCs) for low power generation coupled with industrial waste treatment^6^ and biobatteries for energy storage.^7^ A particularly interesting opportunity for future healthcare applications is sensing EET activity directly from the gut microbiome, considering that pathogenic exoelectrogens have recently been found to colonize the human gut.^8,9^

One of the main directions in EET research is the improvement of the bioelectrical interface of MES, MFC, etc., devices with the aim to increase the signal-to-noise ratio (SNR) of the output signal. The reason for a generally low SNR is a weak EET signal, typically 10–100 fA/cell,^10,11^ produced by only a monolayer of cells formed on the electrode surface.^12^ Materials science offers a particularly versatile array of means to engineer these bioelectrical interfaces. Beyond the commonly used high surface area carbon-based electrode materials such as carbon felt (CF), cloth, and sponge, more complex electrode designs integrate conducting polymers,^13^ redox polymers,^14^ metal oxides,^15^ metal nanoparticles (NPs),^16^ or carbon nanotubes (CNTs) on top of the basic (often carbon) electrode. For example, to artificially increase the number of cells connecting to the electrode, in our previous work, we embedded living bacteria into a 3D biocompatible polymer matrix comprised of poly(3,4-ethylenedioxythiophene):poly(styrenesulfonate) (PEDOT:PSS), resulting in a 20-fold improvement in EET currents.^12^ In another study, we utilized the same material mixed with poly(vinyl alcohol) (PVA) as a charge transducer in amplifying the weak EET signal from a relatively low number of *S. oneidensis* MR-1 cells deposited onto a sub mm^2^ electrode in a transistor configuration.^17^ Another promising approach, intimately combining *S. oneidensis* MR-1 with a graphene oxide-CNT composite, also resulted in enhanced MFC performance.^18^ Despite these and numerous other approaches^19,20^ aiming at improving the bioelectrical interface, studies leveraging the biophysical properties, such as surface charge of the bacterial cell membrane and its extracellular polymeric substances for EET,^21^ have been limited.^22^

Electrostatic attraction between the negatively charged cell membrane of Gram-negative bacteria and positively charged conducting NPs attached to the surface of electrodes has been shown to be a potent method to increase the number of bacteria connected to electrodes. In the medical field, cationic antibacterial peptides have been demonstrated to effectively disrupt the bacterial cell membrane,^23^ or pH-activated polymeric drug-loaded NPs with a positive zeta potential have shown strong multivalent electrostatic-mediated binding with the bacterial cell membrane.^24^ In another study, graphenic materials were covalently functionalized by positively or negatively charged chemical groups to attract or repel *Escherichia coli* cells, respectively.^25^ For electroactive bacteria, functionalization of multiwalled CNTs by positively charged amine-terminated ionic liquids led to an increased number of bacteria on the base carbon electrode along with improved EET currents.^26^ On the other hand, it was reported that the interactions between SWCNTs functionalized by charged NPs and cell membranes do not solely depend on the polarity of charge on the functional group. For example, researchers succeeded in the delivery of genes into chloroplasts by utilizing the penetration of chitosan-complexed SWCNT carriers through the cell membrane.^27^ A key feature of this approach seemed to be the magnitude of the zeta potential of the functionalized SWCNTs, i.e., higher values (>44 mV) led to penetration while medium to lower values (<35 mV) led to no penetration through the cell membrane, regardless of the polarity.^28^ Therefore, in addition to the conducting polymers mentioned above and various NPs,^29^ SWCNTs are another promising material system for bioelectrical interfacing thanks to their high electrical conductivity, mechanical strength, and optical absorption and emission in the IR spectrum. Moreover, the attachment of various functional chemical groups to SWCNTs can potentially enhance their connection to the bacterial cell membrane.^30^ While several studies have reported on the use of SWCNTs for bacterial-electrode interfaces,^31,32^ only a few studies^26^ investigate CNTs functionally matched to the biophysical environment of the exoelectrogenic cell membrane to improve EET.

In this study, we modified SWCNTs using positively or negatively charged or neutral ligands and investigated the electrochemical performance of *S. oneidensis* MR-1 in MESs where we introduced these SWCNTs. To test the compatibility of this approach for zeta potentials of various magnitudes and functional groups of various sizes, we prepared and characterized SWCNTs wrapped by positively or negatively charged cellulose derivatives or attached by small-sized positive or neutral surfactants, respectively. By mixing the aqueous SWCNT solutions into MESs we observed two distinct behaviors based on the ligand used: (i) continuously increasing EET currents in the case of SWCNTs wrapped by a polysaccharide derivative, irrespective of their charge, and (ii) a decrease in the signal in the case of SWCNTs functionalized by small-sized surfactants, compared to control MESs not containing SWCNTs. The origin of these changes was investigated using chronoamperometry, cyclic voltammetry, and electrochemical impedance spectroscopy techniques, while the interaction of bacteria with the electrode was investigated by visual and microscopic observations. We believe that our approach brings value to the field by using the physical properties of the bacterial cell membrane itself as the starting point for developing the bioelectrical interface and by utilizing functional NPs based on SWCNTs, easily tunable in their chemical and physical properties.

## 2 Experimental Section

### 2.1 Solubilization of SWCNTs

Powders of SWCNTs (high-pressure carbon monoxide, HiPco) were dispersed in water containing each dispersant (hydroxyethylcellulose ethoxylate, quaternized (HEQ) 0.10 wt %, carboxymethylcellulose sodium salt (CMCNa) 0.10 wt %, hexadecyltrimethylammonium chloride (CTAC) 1.0 wt %, or t-octylphenoxypolyethoxyethanol (Triton X) 1.0 wt %) through sonication using bath-type and microtip sonicators. The resulting dispersions were ultracentrifuged at 100,000 relative centrifugal force (RCF) for 0.5 h to remove SWCNT aggregates, and then the supernatant (top 70%) was collected for spectroscopic characterizations, as described in Section 2.2. Excess free polymers were removed from the SWCNT-HEQ and SWCNT-CMCNa dispersions by collecting the HEQ- and CMCNa-wrapped SWCNTs, respectively, via ultracentrifugation and redispersion in pure water by sonication. This procedure could not be applied to CTAC and Triton X because the removal of the excess surfactants induced aggregation of SWCNTs under low concentration conditions.

### 2.2 Spectroscopic Characterizations of SWCNTs

Ultraviolet–visible-near-infrared (UV/vis/NIR) absorption spectra were measured using a V-670 (JASCO). Photoluminescence (PL) spectra and 2D PL mapping images were taken by using a HORIBA Jobin Yvon spectrofluorometer (FluorologR-3 with FluorEssence). The optical measurements were conducted at room temperature (∼20 °C) by using quartz cells with a path length of 0.20 cm. The concentrations of the final solutions were determined based on absorbance values at 1137 and 1134 nm, corresponding to (7,6) chirality, as *A*_1137_ = 0.221 for SWCNT-HEQ, *A*_1132_ = 0.679 for SWCNT-CMCNa, *A*_1134_ = 0.999 for SWCNT-CTAC, and *A*_1142_ = 0.904 for SWCNT-Triton X.

### 2.3 Zeta Potential Measurement of SWCNTs and Bacteria

All SWCNT solutions were prepared by the procedure described above for measurements. For bacteria, stock solutions of wild-type (WT) *S. oneidensis* MR-1 were dissolved in deionized water to concentrations of optical density at 600 nm (OD_600_) = 0.1 and thoroughly vortexed. Zeta potentials were measured with a Zetasizer Nano ZS90 (Malvern) in a disposable capillary cell DTS1070 with *n* = 6 repetitions and a measurement time of 120 s.

### 2.4 Strains and Growth Conditions

Wild-type *S. oneidensis* MR-1 and its green fluorescent protein (GFP)-expressing mutant were generously donated by the group of Prof. Caroline M. Ajo-Franklin. The GFP-expressing strain carried a plasmid encoding *gfp* and a kanamycin-resistance gene, enabling visualization by fluorescence microscopy.^33^ Cultures were inoculated from frozen glycerol stocks into two 250 mL sterile Erlenmeyer flasks, each containing 50 mL of Luria–Bertani (LB, Sigma-Aldrich) broth, and grown overnight at 30 °C with 110 rpm shaking to an average OD_600_ = 1.7 and 2.2 for WT and GFP, respectively. After overnight growth, the cells were harvested by centrifugation (3–18 K, Sigma) at 4000 RCF at 4 °C for 10 min and washed then resuspended twice with M9 minimal salts medium (M9, Sigma-Aldrich). The first washing was followed by one more centrifugation step with the same parameters as above. Finally, the combined cell pellet was resuspended in 4 mL of the M9 medium resulting in 25-fold concentrated stock suspensions. Bacteria were handled under sterile conditions. Cultures of *S. oneidensis* MR-1 expressing GFP were grown with 50 μg/mL kanamycin (Sigma-Aldrich). We note that the growth rates for the WT and GFP somewhat differed; therefore, we used different volumes of the concentrated stock suspensions for the experiments of each to reach the same OD_600_ = 0.26.

### 2.5 Preparation of MESs

Borosilicate glass vials filled with M9 (100 mL), open GL45 screw caps fitted by PTFE-coated silicone septums, Ti (99.99%, diameter 0.5 mm, Alfa Aesar), and Pt (99.95%, diameter 0.5 mm, Alfa Aesar) wires were autoclaved 1 day prior to experiments in an Elara 9i autoclave (Tuttnauer) and stored in sterile conditions. Carbon felt (Alfa Aesar, 99%, thickness 6.35 mm) was precut by a hole cutter to a cylindrical shape with a diameter of 12 mm to yield 0.72 cm^3^ geometric volume and 4.66 cm^2^ geometric surface area. The CF acted as a working electrode (WE) vs an Ag/AgCl wire reference electrode (RE), while a Pt wire was used as a counter electrode (CE). The WE was connected to the potentiostat by a Ti wire. Prior to experiments Ag/AgCl wires were cleaned as follows: residual AgCl layer was cleaned off the base Ag wire with fine-grain sandpaper and wiped with clean paper wetted by isopropyl alcohol and then deionized (DI) water. Then, the AgCl layer was regenerated by immersion into a FeCl_3_ solution (1 M) for ∼10 min, followed by wiping with isopropyl alcohol and DI water before each experiment. Lactate solution (stored at 4 °C and filter-sterilized) was added to the sterile M9 (100 mL) in MESs in the vicinity of a flame from a Fireboy Plus Bunsen burner (Integra) to 40 mM. Ti wires pierced through the CF, Ag/AgCl, and Pt wires were introduced into the MESs through the silicone septum near the flame of the Bunsen burner to maintain sterile conditions. CF/Ti WEs and Ag/AgCl wires were flushed with ethyl alcohol for sterilization followed by DI water immediately before insertion into MESs. During this flush-sterilization process, CF WEs were lightly compressed and released several times by gloved hand to remove trapped air bubbles. Aqueous SWCNT solutions were introduced into the MESs to final concentrations of *A*_1137_ = ∼6.65 × 10^–3^ through sterile needles before the start of recording. N_2_ gas was purged into the MESs and mechanical stirring was applied by stir bars for the duration of the experiments. Bacteria were added into the MESs after a series of abiotic measurements including at least 30 min of chronoamperometry (CA) recording. The average ambient temperature in the vicinity of the MESs was 23.4 ± 0.7 °C (standard deviation).

### 2.6 Electrochemical Techniques

For all experiments, Interface 1010B potentiostat/galvanostats were used and controlled by the Gamry Framework software (both from Gamry Instruments). Electrochemical measurements including chronoamperometry, cyclic voltammetry, and electrochemical impedance spectroscopy were performed in an automated sequence with no waiting times between the techniques to maintain a constant potential on the WE (except for cyclic voltammetry), the latter providing the necessary oxidizing potential for EET. The sequence of the program was as follows: open circuit voltage measurement → differential pulse voltammetry (DPV, abiotic, not discussed in this manuscript) → cyclic voltammetry (CV, abiotic) → electrochemical impedance spectroscopy (EIS, abiotic) → CA (added bacteria after ≥30 min) → EIS → DPV (not discussed in this manuscript) → CV. In addition, to account for statistical variations, 4–5 MESs were controlled and monitored simultaneously, each with a standalone Interface 1010B but the same sequence program. In all cases, nitrogen gas was directly purged into the M9 solution containing 40 mM lactate (sodium L-lactate, Sigma-Aldrich). For CA, +0.2 V was applied on the WE vs RE. CVs were performed with a scan speed of 10 mV/s; four cycles were done for each experiment, and the fourth cycle was used for data plotting. EIS was performed with a stimulus of 10 mV peak-to-peak on a +0.2 V_Ag/AgCl_ DC voltage in the frequency range from 20 kHz to 1 mHz.

### 2.7 EIS Modeling

The equivalent circuit model (Figure S1) was constructed via the Impedance Model Editor of Gamry Echem Analyst software, and impedance fitting was performed by the Simplex Method of the same software. The goodness of fit values for impedance fitting and for Kramers–Kronig analyses were on the order of 10^–4^ and 10^–6^, respectively; the fitted curves are shown in Figure S2. A low number indicates a low deviation between the measured and the simulated data points.

### 2.8 Microscopy

After CA the measurement sequence was terminated, the CF WEs were detached and gently rinsed in sterile M9 three times, each time in a new M9 solution and finally in deionized water. Imaging was done on a Ni-E upright motorized microscope (Nikon), equipped with a Zyla sCMOS camera (Andor Technology). The illumination and excitation source was a Lambda DG-4 ultrahigh-speed wavelength switching illumination system (Sutter Instrument), introduced from the top side. The area of observation was precisely selected by controlling a microscope stage in the *x*-*y* directions using a ProScan III universal microscope automation controller (Prior). Different magnifications were achieved by manually switching between a Fluor WD 2.0 60×/1.00 DIC H/N2 water dipping objective and a Plan Fluor WD 0.16 100×/1.30 OFN25 DIC H/N2 oil immersion objective (both from Nikon). For imaging, the 60× objective was dipped into a glass Petri dish containing M9 and the CF WE, while in the case of the 100× objective through a refractive index-matching oil layer applied onto a coverslip, the latter was placed on top of the wet CF WE. The microscope was equipped with bright field and FITC filter sets, the latter for fluorescence imaging.

## 3 Results and Discussion

### 3.1 Preparation and Characterization of Functionalized SWCNTs

**Figure 1:**
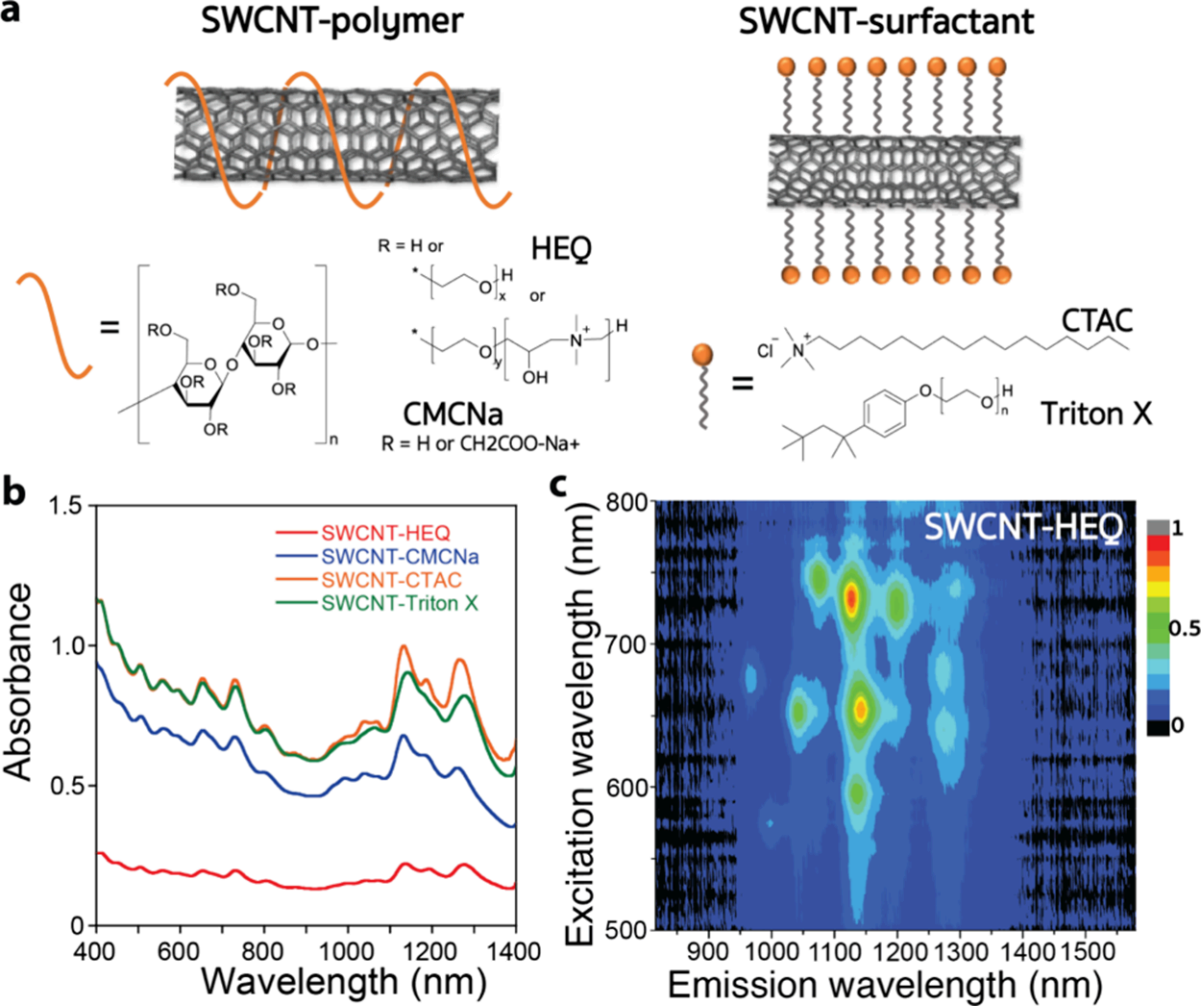
Functionalized SWCNTs used in this study and their optical characterization. (a) Chemical structures of HEQ, CMCNa, CTAC, and Triton X, and schematic images of the functionalized SWCNTs with polymer wrapping and surfactant coating. (b) UV/vis/NIR absorption spectra of solubilized SWCNTs using HEQ, CMCNa, CTAC, and Triton X. Differences in amplitudes are attributed to differences in concentrations. (c) 2D PL mapping image of SWCNT-HEQ.

To endow SWCNTs with a positive or negative charge, as well as make them water-soluble, we used three kinds of dispersants for functionalization (Figure 1a): the surfactant hexadecyltrimethylammonium chloride (CTAC), a typical SWCNT dispersant,^34^ and the polymeric dispersants hydroxyethylcellulose ethoxylate, quaternized (HEQ; positively charged) and carboxymethylcellulose sodium salt (CMCNa; negatively charged). HEQ and CMCNa are cellulose derivatives with hydrophilic charged side chains promoting water solubility and a hydrophobic main chain that can interact with the SWCNT surface and have been found to solubilize SWCNTs individually.^35^ In addition, polymeric dispersants wrap SWCNTs in a stable coating fashion, which is different from the dynamic micellar coating of small surfactant CTAC (Figure 1a). Thus, stable surface functionalization is expected for the polysaccharide-wrapped SWCNTs. As a control, we also prepared SWCNTs functionalized by the neutral surfactant t-octylphenoxypolyethoxyethanol (Triton X, Figure 1a).

Powders of SWCNTs were dispersed in water containing each dispersant at concentrations below 1 wt % through sonication processes (details are described in Section 2). The resulting dispersions were ultracentrifuged to remove SWCNT aggregates, and the supernatant was collected for spectroscopic characterizations (UV/vis/NIR absorption and 2D PL), as described below. In addition, excess free HEQ polymers that might act as antimicrobial agents were removed from the solution through the collection of the HEQ or CMCNa polymer-wrapped SWCNTs by ultracentrifugation and redispersion in pure water by sonication. From here on, we refer to the resulting functionalized aqueous dispersions of SWCNTs as SWCNT-HEQ, SWCNT-CTAC, SWCNT-CMCNa, and SWCNT-Triton X.

In well-solubilized SWCNTs, clear absorption peaks can be observed that are related to well-defined chiralities. Figure 1b shows the UV/vis/NIR absorption spectra of the SWCNT solutions prepared by using each dispersant. For each of the SWCNTs solubilized by the various functional groups, obvious peaks were observed in the NIR region, corresponding to chiralities of (8,3), (6,5), (7,5), (10,2), (7,6), (8,6), and (9,5) (the corresponding wavelengths are listed in the Supporting Information).^36^ Small wavelength shifts between each material can be attributed to the differences in the surrounding microenvironments constructed by wrapped or attached dispersants.^37^ Even using different dispersants, the chirality distribution of the solubilized SWCNTs was the same across different samples, meaning that the prepared solutions had identical and well-solubilized SWCNT components. Furthermore, the photoluminescence (PL) of SWCNTs can be observed only from individually solubilized SWCNTs because bundled SWCNTs suffer from intertube energy transfer that causes PL quenching.^38^ Therefore, we used 2D PL mapping on all obtained solutions to detect individually solubilized SWCNTs more precisely with high sensitivity. In Figures 1c and S3, the vertical and horizontal axes correspond to excitation and emission wavelengths (λ_ex_ and λ_em_). In addition to the chiralities observed by the UV/vis/NIR technique described above, Figure 1c reveals PL from SWCNT-HEQ with chiralities (8,4), (10,3) (9,4), and (8,7) (the full list of chiralities and the corresponding wavelengths are listed in the Supporting Information).^39^ The other three functionalized SWCNTs showed almost the same PL features, indicating that the components of individually solubilized SWCNTs were identical (Figure S3). Therefore, SWCNT solutions containing individually solubilized tubes were successfully prepared for the interaction with bacteria.

To facilitate electrostatic attraction of the bacterial cell membrane, we expect the zeta potentials of the solubilized SWCNTs to be in the same range as that of the bacteria. The zeta potentials were measured to be +28.48 ± 1.44 mV for SWCNT-HEQ, +51.69 ± 0.78 mV for SWCNT-CTAC, −64.83 ± 1.53 mV for SWCNT-CMCNa, and −26.06 ± 2.67 mV for SWCNT-Triton X, from which we confirmed that the surface charges of the functionalized SWCNTs changed depending on the dispersants. The zeta potential of *S. oneidensis* MR-1 (wild type, WT) was measured to be −37.2 ± 11 mV and we therefore expect electrostatic interactions with charge-functionalized SWCNTs in MESs.

### 3.2 Polymer-Wrapped SWCNTs Enhance EET Currents of *S. oneidensis* MR-1

To gain insights into the interactions of bacteria with the newly prepared functionalized SWCNTs and how these interactions influence the electrochemical performance of MESs, we employed a variety of electrochemical techniques. Chronoamperometry (CA) is one of the most widely used techniques to monitor the electrochemical activity of exoelectrogenic bacteria by collecting a flow of charges over time at a fixed potential. For this experiment, we applied a constant potential of +0.2 V_Ag/AgCl_ on the porous carbon felt (CF) working electrode (WE) versus a Ag/AgCl wire reference electrode (RE) to provide the necessary oxidizing potential for EET. The employed single compartment MESs also contained a platinum wire counter electrode (CE, Figure S4) M9 minimal medium electrolyte supplemented by lactate (40 mM), bacterial suspension (WT *S. oneidensis* MR-1, optical density at 600 nm (OD_600_) of 0.26), and, when applicable, an aqueous solution of functionalized SWCNTs.

**Figure 2:**
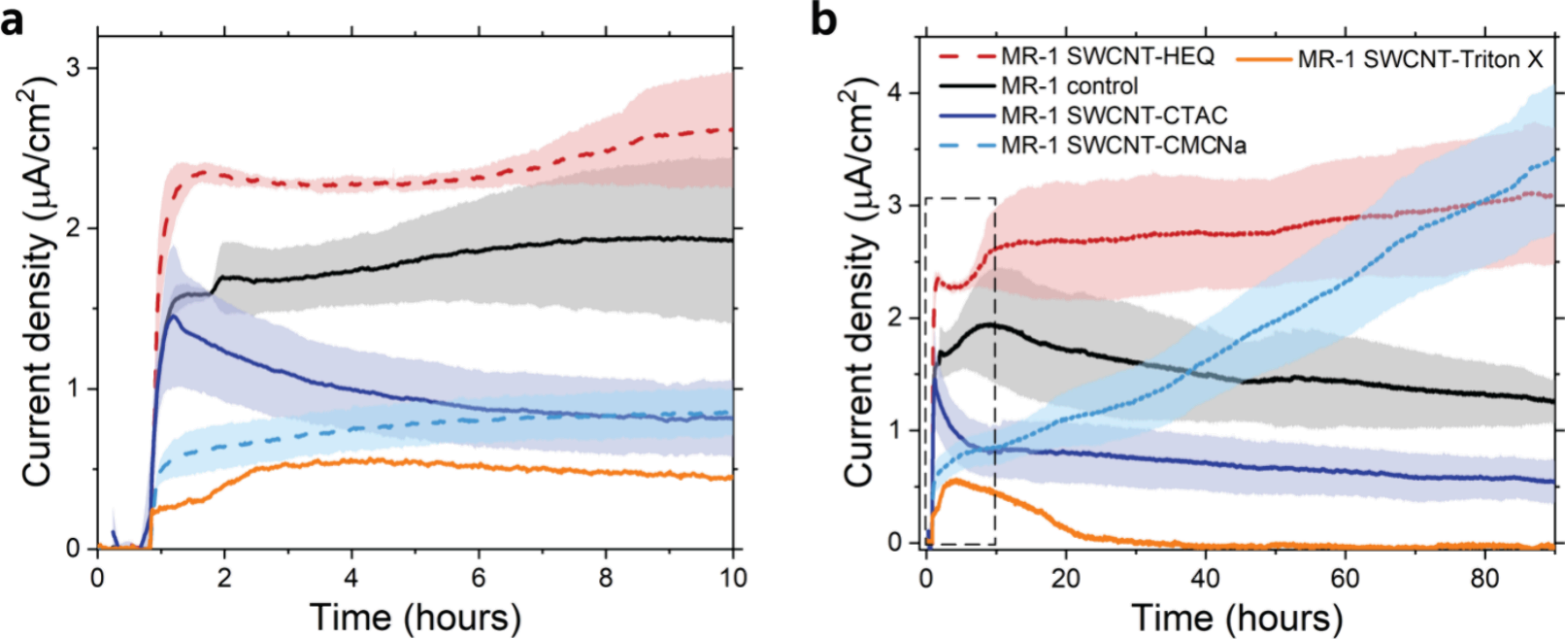
Chronoamperometric currents of *S. oneidensis* MR-1 bacteria (OD_600_ = 0.26) in the presence of SWCNTs functionalized by HEQ (dashed red lines), CTAC (blue line), CMCNa (dashed light blue lines), Triton X (orange lines) and no SWCNTs (control, black lines) present, in anoxygenic environment in M9 electrolyte with lactate (40 mM) at +0.2 V_Ag/AgCl_. (a) Zoom over the first 10 h and (b) 90 h of CA recordings. Solid lines are mean, and translucent ribbons are standard deviation (SD) for *n* = 3 measurements in each case, except for SWCNT-Triton X, which is from one recording. Dashed square in *b* highlights the zoomed-in part shown in *a*.

CA measurements of MESs without SWCNTs, termed “MR-1 control”, exhibited a relatively fast initial rise in currents after the insertion of bacteria into the MESs already containing lactate, followed by a slower period of increase over approximately 8 h reaching a peak current density of *I*_max_ = ∼1.9 μA/cm^2^ (Figure 2a). Thereafter, we observed a trend of a slow decrease reaching ∼1.3 μA/cm^2^ after ∼90 h (Figure 2b). We believe that this trend does not relate to growth conditions because, first, there were no growth factors in the MESs,^12^ and second, we did not observe changes in OD_600_ of the bulk solution (during and after CA) that could be plausibly correlated with the CA (Figure S5). Therefore, the above data must mainly reflect the self-attachment and stabilization process of bacteria onto the CF fibers, as well as the production of flavin-based soluble electron shuttles. We observed surprising changes in the CA data when aqueous suspensions of functionalized SWCNTs were introduced into the MESs, preceding the injection of bacteria. Current levels in MESs with either positively or negatively charged cellulose-based SWCNTs continuously increased over the 90 h of monitoring (discussed further below), except for the initial ∼10 h that resembled the trend of “MR-1 control” (Figure 2). In addition, current levels were higher and lower in this initial period for “MR-1 SWCNT-HEQ” and “MR-1 SWCNT-CMCNa”, respectively, compared to “MR-1 control”. The low initial levels of “MR-1 SWCNT-CMCNa” might at least partially be explained by batch-to-batch variations in the electrochemical performance of *S. oneidensis* MR-1 (Figure S6). On the other hand, MESs containing small surfactant-functionalized SWCNTs exhibited an inferior behavior compared to the “MR-1 control”. Soon after reaching an initial peak, “MR-1 SWCNT-CTAC” displayed a strong decrease in current levels for the first 7 h (Figure 2a), thereafter having a slow decrease similar to “MR-1 control”. Interestingly, current levels of “MR-1 SWCNT-Triton X” diminished after approximately 20 h of operation after an initial short increase. For this experiment, unlike the other cases, we added SWCNTs into the MES *after* bacteria injection because our initial observations showed zero current when SWCNT-Triton X preceded the introduction of bacteria into the MES. The current levels at 90 h were ∼1.3 μA/cm^2^ for “MR-1 control”, 3.1 and 3.4 μA/cm^2^ for “MR-1 SWCNT-HEQ” and “MR-1 SWCNT-CMCNa”, while only ∼0.6 and 0 μA/cm^2^ for “MR-1 SWCNT-CTAC” and “MR-1 SWCNT-Triton X”, respectively (Figure 2b).

The lower currents of “MR-1 SWCNT-CTAC” compared to “MR-1 control” likely arose from the detachment of CTAC surfactants causing the aggregation of SWCNTs soon after introducing them into MESs, even before polarizing the WE, as evident in Figure S4. Indeed, it has been shown that decreasing the residual surfactant concentration in the solution results in the aggregate formation of SWCNTs.^40^ In some cases, such an effect might have caused the decrease in OD_600_, as we measured after the end of CA (Figure S5), likely contributing to the lower current levels with SWCNT-CTAC. Aggregated SWCNT-CTAC tended to adhere to all electrode surfaces, including RE and CE, as opposed to, for example, SWCNT-HEQ which adhered primarily to the CF WE in a smoother fashion (Figure S4). On the other hand, no decrease was observed in OD_600_ for MESs containing SWCNTs functionalized with cellulose derivatives (Figure S5). For “MR-1 SWCNT-Triton X”, we measured a greatly reduced OD_600_ after the end of CA compared to the initial value, but no such extensive aggregation of SWCNTs was visible as compared to SWCNT-CTAC (Figure S4). This result suggests that, unlike CTAC, Triton X may not be detaching from SWCNTs, and thus, SWCNT-Triton X itself may be detrimental to bacterial cells. Finally, we should also consider the case when not all surfactants are detached from the SWCNTs. Compared with SWCNT-HEQ, SWCNT-CTAC showed a higher positive zeta potential and somewhat blue-shifted SWCNT absorption peaks. These results indicate a dense covering of CTAC molecules on the tube surface^34,41^ that could interfere with electron transport between SWCNTs and bacterial cells. Therefore, the observed high performance of SWCNT-HEQ could also be partially due to the moderate coating of HEQ on the SWCNTs. This would allow not only cationic functionalization for cell complexation but also enhanced electron transport via partially bare tube surfaces in proximity to bacterial cells. In summary, we observed that both positively and negatively charged cellulose-functionalized SWCNTs are interesting candidates to enhance EET currents of *S. oneidensis* MR-1 with an unusual continuously increasing trend over the course of the CA. Meanwhile, small surfactant-functionalized SWCNTs led to lower or diminished EET currents compared to the biotic control either due to surfactant detachment-induced aggregation of SWCNTs or detrimental effects on the bacteria.

### 3.3 Cyclic Voltammetry Indicates Different Mechanisms of Current Enhancement Depending on the Charge of Polymer-wrapped SWCNTs

**Figure 3:**
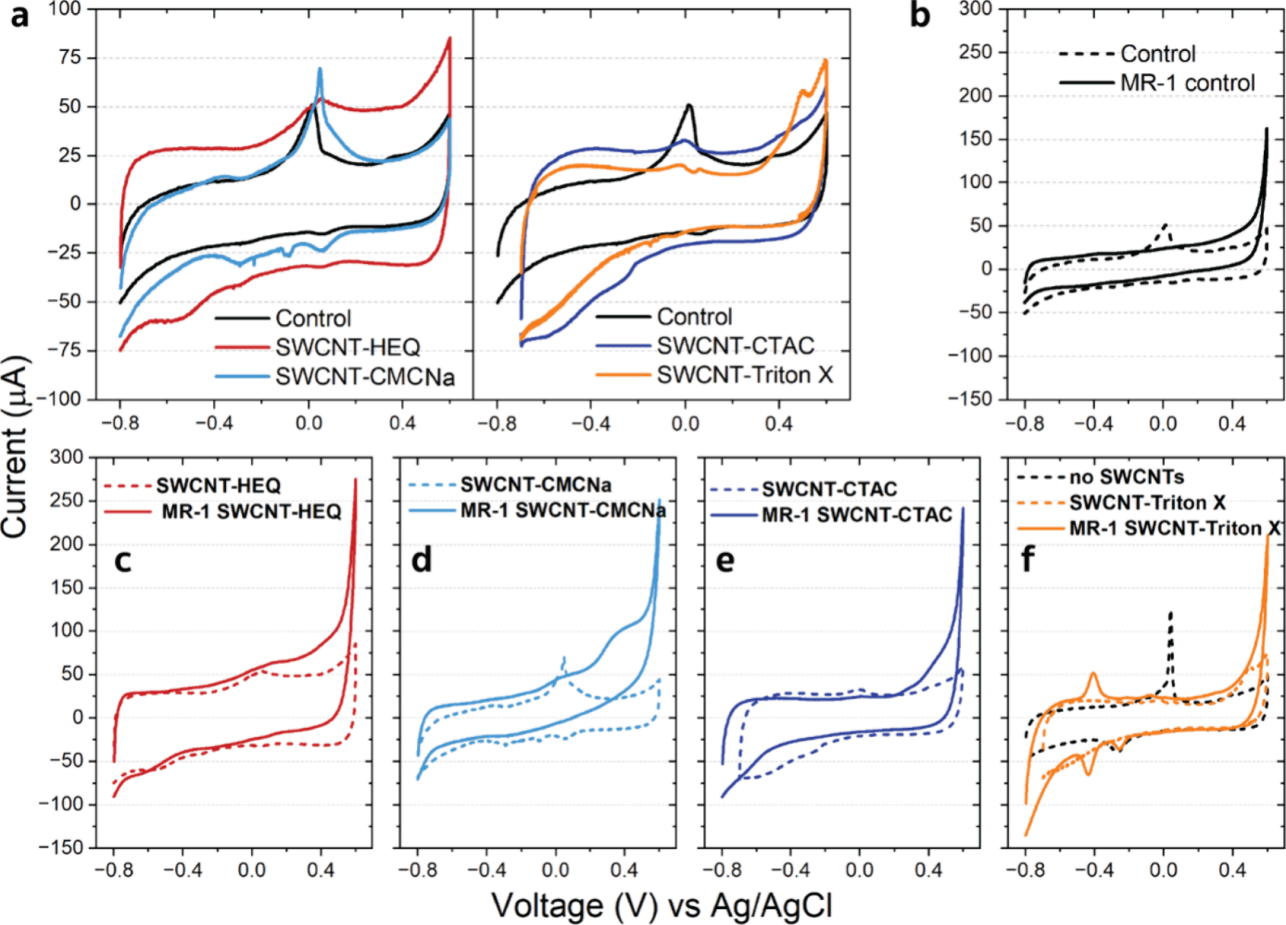
(a) Comparisons of abiotic cyclic voltammograms measured in bacterial reactors without (Control) and in the presence of SWCNTs functionalized by various chemical groups and (b–f) abiotic–biotic CV pairs with (solid lines) and without (dashed lines) *S. oneidensis* MR-1 (OD_600_ = 0.26) when (b) no SWCNTs are present and (c–f) in the presence of SWCNTs functionalized by various groups in anoxygenic environment in the M9 electrolyte with lactate (40 mM), at scan speed 10 mV/s. Abiotic data were acquired immediately before abiotic EIS, while biotic data were acquired soon after biotic EIS, following chronoamperometry.

Cyclic voltammetry (CV) can reveal typical signatures of EET, as well as possible increases in the electrochemically active surface area (EASA) of the WE by SWCNTs. We performed CVs before and after the chronoamperometric recordings on all experimental variations, shown in Figure 3. Representative abiotic CVs indicated an increase in capacitive currents by SWCNT-HEQ and SWCNT-CTAC compared to bare CF, from 21.4 to 48.8 and 28.6 μA (∼2.3-fold and ∼1.3-fold, respectively), while only a slight increase and even a small decrease when adding SWCNT-CMCNa (to 22.4 μA) and SWCNT-Triton X (to 17.7 μA, i.e., ∼0.8-fold change), shown in Figure 3a and summarized in Supporting Information Table S1. We determined these values at +0.3 V_Ag/AgCl_ on the cathodic side where the abiotic CVs appear to be largely unaffected by noncapacitive electrochemical exchange currents and charging processes. Changes in capacitive currents are indicative of proportional changes (enlargement or reduction) in EASA of the WEs due to the adhesion of conducting SWCNTs to CF in the case of SWCNT-HEQ and SWCNT-CTAC, while presumably a partial coverage of the CF surface by nonconducting surfactants only in the case of SWCNT-Triton X. Indeed, the increase in maximum current levels of “MR-1 SWCNT-HEQ” compared to “MR-1 control” in the CA in the initial ∼10 h (Figure 2a) agrees with the higher capacitive currents of SWCNT-HEQ versus bare CF (control). From these data and Figure S4, we conclude that in the case of SWCNT-HEQ the adhesion of SWCNTs to CF first occurred in abiotic MESs with nonpolarized WEs and then remained on the CF regardless of the voltage bias during CV. On the other hand, lower capacitive currents for the other three electrodes point toward lower utilization of SWCNTs. This is likely the result of aggregation and simultaneous adhesion to surfaces other than just the WE in the case of SWCNT-CTAC, as described above, while either nonadherence of SWCNTs to CF or coverage of CF by nonconducting ligands in the case of SWCNT-CMCNa and SWCNT-Triton X, respectively (Figure 3a).

Increased anodic currents in biotic MESs compared to abiotic ones are typically a signal of EET. For “MR-1 control”, such an increase at +0.2 V_Ag/AgCl_ (the same voltage was used for CA) was relatively low (Figure 3b), corresponding to the moderate signal level of 28.6 μA measured at the end of CA (Figure 2b). Specific current values extracted from CVs for all SWCNT versions are summarized in Supplementary Table S1. In the case of “MR-1 SWCNT-HEQ” (Figure 3c) and ‘MR-1 SWCNT-CMCNa’ (Figure 3d) which had the highest CA signals at 90 h, the CV curves revealed significantly higher values of 65.4 and 59 μA, respectively, taken at the same voltage. After subtracting the abiotic data from the biotic data at +0.2 V in the CVs, we can see that the EET-only signal of “MR-1 SWCNT-HEQ” was only half (17.1 μA, Table S1) of that of “MR-1 SWCNT-CMCNa” (34 μA), reflecting the higher signals of “MR-1 SWCNT-CMCNa” measured in the CA data. Therefore, SWCNT-CMCNa leads to an increase in CA currents over that of SWCNT-HEQ even without a change in EASA. On the other hand, there was no noticeable deviation in the CV at +0.2 V_Ag/AgCl_ for “MR-1 SWCNT-CTAC” from its abiotic counterpart (Figure 3e), which can partly be explained by low EET current signals observed in the CA. Finally, “MR-1 SWCNT-Triton X” surprised us with a somewhat higher value of 24.2 μA compared to its abiotic counterpart (Figure 3f), even though at the time of data acquisition, its CA signal had already been negligible (Figure 2b). While we cannot point to the origin of this small signal, the presence of another anodic–cathodic signal peak pair close to −0.4 V_Ag/AgCl_, appearing only for “MR-1 SWCNT-Triton X”, likely indicates the presence of another non-EET electroactive or adsorbing/desorbing species at the CF surface.

Next, we investigated possible causes of the continuously increasing CA currents shown in Figure 2b for “MR-1 SWCNT-HEQ” and “MR-1 SWCNT-CMCNa”. A plausible explanation could be a continuous and very slow accumulation of cellulose-based SWCNTs on the surface of the CF after the initial attachment, discussed above (and Figure S4), in turn allowing more bacteria to attach to the electrode. However, this must not be the case because there was no clear enlargement in the EASA for either SWCNT-HEQ and SWCNT-CMCNa from before to after the CA process, indicated by the largely overlapping cyclic voltammograms of the biotic and abiotic data sets (not considering the contributions of EET and any other exchange currents) shown in Figure 3b. These observations suggest a possible gradual and slow attachment of bacteria to the CF, rather than both bacteria and SWCNTs, over the course of the CA when using SWCNTs functionalized with cellulose derivatives, which in turn enhance EET currents.

In summary, while the positively charged surfactant-functionalized SWCNTs somewhat increased the EASA through the attachment of aggregated SWCNTs to CF, they did not enhance EET due to the detrimental effect on bacteria. Adding neutral surfactant SWCNTs slightly decreased the EASA of CF presumably through its coverage by Triton X, while the presence of an anodic–cathodic peak pair at around −0.4 V after the biotic CA points to an electroactive process presumably related to the detrimental effect of Triton X on bacteria, corroborating the low OD_600_ value shown in Figure S5. Positively charged cellulose-based SWCNTs significantly increased the EASA of CF, resulting in a total increase in the EET currents. On the other hand, negatively charged cellulose-based SWCNTs induced a substantial increase in the EET signature of CV without a notable change in the EASA of CF, indicating that these SWCNTs did not attach to the CF.

### 3.4 Fluorescence Microscopy Indicates a High Density of *S. oneidensis* MR-1 on Carbon Felt Electrodes in Polymer-Wrapped SWCNT-Based MESs

Based on the results of CA and CV, we expect (i) a high number of bacteria on CFs blended with cellulose-functionalized SWCNTs and (ii) the presence of SWCNTs on CF especially for “MR-1 SWCNT-HEQ” due to the enlarged EASA. We first compared photographs of abiotic and biotic CFs (i.e., before and after electrochemical measurements) in MESs without any SWCNTs added, and in samples with expected attachment of SWCNTs to CF (SWCNT-HEQ and SECNT-CTAC). Visual observations of WEs still immersed in MESs revealed light brown thick hair-like structures for “MR-1 control” which were not present on abiotic “Control” CF WEs (Figure S7). We assume that these structures are biofilms of *S. oneidensis* MR-1 that grew on the surface and in the pores of the CF, including on thin CF hairs protruding from the surface. The presence of SWCNT-HEQ in abiotic MESs led to the attachment of SWCNTs to CF as thin black hair-like structures (Figure S4), while after CA with bacteria, these hairy structures appeared much denser (Figure S7), indicating the presence of biofilms on SWCNT-HEQ hairs. Finally, with SWCNT-CTAC present, both abiotic and biotic MESs showed strong aggregation of SWCNTs and attachment to all electrode surfaces with no clear differences between before and after recording EET currents (Figures S4 and S7). These observations suggest that *S. oneidensis* MR-1 can attach readily to bare CF in significant numbers, while SWCNTs remained attached to both the SWCNT-HEQ/CF and SWCNT-CTAC/CF electrodes even after the lengthy CA recordings in the presence of bacteria.

**Figure 4:**
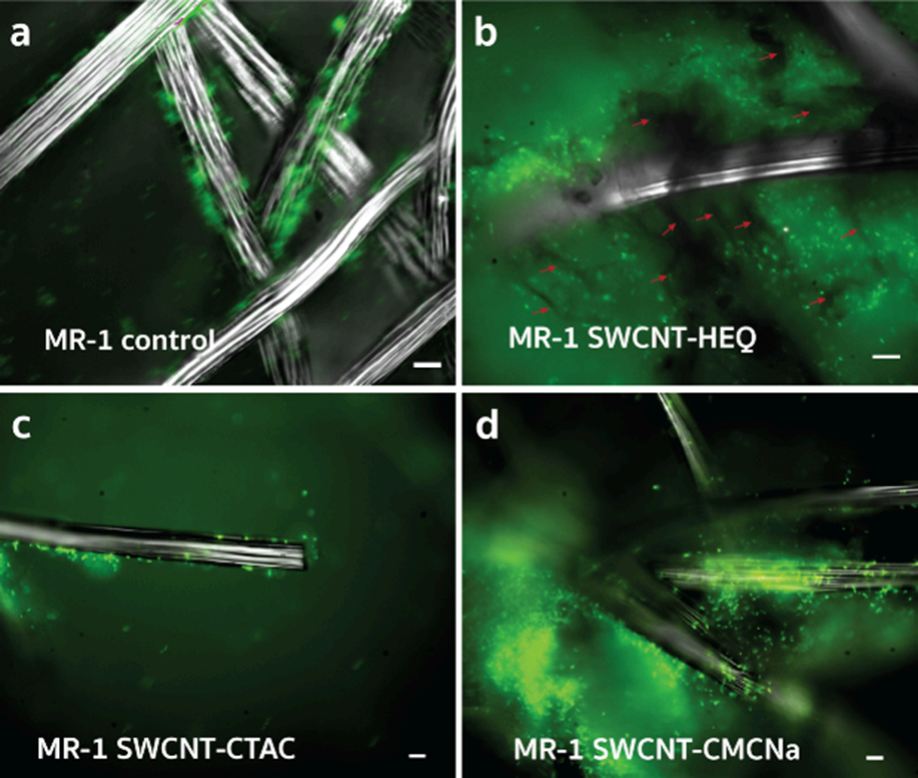
Microscopic observations in the presence of *S. oneidensis* MR-1 and SWCNTs. Combined bright field and fluorescence FITC microscopy images (GFP mutant, OD_600_ = 0.25) of carbon felt electrodes (a) without and (b–d) with chemically functionalized SWCNTs. Green color marks the GFP mutant, wide silver rod-like structures are CF, and thin black threadlike structures and areas (some of them marked by red arrows) in *b* are assumed to be weak aggregates of SWCNTs. Scale bars are 10 μm. “MR-1 control”, “MR-1 SWCNT-HEQ”, and “MR-1 SWCNT-CMCNa” images were taken after ∼91, ∼80, and ∼100 h of chronoamperometry, respectively.

To obtain a more detailed image of bacteria on CF, we observed the surface of CF samples without and with SWCNT-HEQ, SWCNT-CMCNa, and SWCNT-CTAC after CA by fluorescence microscopy, employing a GFP mutant of *S. oneidensis* MR-1 injected at the same OD_600_ as the WT. The GFP mutant is nearly identical to the WT strain, except for the expression of green fluorescent proteins for imaging purposes. For “MR-1 control” we identified three main types of regions: (i) monolayers of bacteria on the surface of CF fibers (Figure 4a), also observed in other studies;^12^ (ii) biofilms closely surrounding single CF fibers (Figure S8aI); and (iii) larger biofilms in spaces between CF fibers (Figure S8aII,III). We note that based on our observations, the distribution of bacteria throughout the surface and pores of CF is nonuniform, which may also be related to the circular flow of electrolyte solution induced by stirring. For the “MR-1 SWCNT-HEQ” samples, in addition to GFP bacteria, we also observed thin black hairs surrounding the much thicker CF fiber bundles that we assume to be weakly aggregated SWCNT-HEQ fibers (Figures 4b and S8b). GFP bacteria seem to be located around these SWCNT-HEQ hairs in large numbers, where the SWCNT-HEQ presumably provides an extended surface area for the mutant. Because GFP was found only scarcely on the surface of CF fibers, we suggest that SWCNTs in contact with both CF and bacteria would let more *S. oneidensis* MR-1 be conductively linked to CF compared to the “MR-1 control”, which is reasonable due to the presence of the positively charged HEQ polymer around the SWCNTs. For “MR-1 SWCNT-CTAC”, we were able to confirm the presence of a relatively low number of GFP mutants on CF bundles (Figure 4c). However, after repeated attempts, we were not able to detect the black hairy structures or extended biofilms between the CF bundles. This may be a limitation of our method. Finally, the “MR-1 SWCNT-CMCNa” samples revealed a considerably large number of GFP mutants on both CF bundles and in their gaps (Figures 4d and S8c), but not the presence of black hairy structures resembling SWCNTs, in accordance with the CV and CA results.

In conclusion, the increased EASA provided by SWCNT-HEQ combined with the observed affiliation between *S. oneidensis* MR-1 with black hairy structures (assumed to be weakly aggregated SWCNT-HEQ), gives a reasonable explanation for the enhanced and continuously increasing EET currents observed in Figure 2b. On the other hand, a similar continuous increase observed for “MR-1 SWCNT-CMCNa” in the CA is linked to a large number of bacteria surrounding CF bundles, but without growth in the bulk electrolyte (Figure S5) and without the presence of SWCNTs on the CF. While positively charged SWCNT-HEQ is expected to attract negatively charged bacterial membranes, leading to enhanced EET, an opposite effect between negatively charged SWCNT-CMCNa and bacteria may not hold true based on our observations. In a scenario where CMCNa would partly detach from SWCNTs and attach to CF, a possible gradual protonation of the carboxylate anion in CMCNa due to a gradually decreasing pH in the microenvironment of biofilms (due to metabolism) could occur. Such protonation would neutralize the negative charge on CMCNa, leading to a gradually decreasing Coulombic repulsion between the carboxylic groups and bacterial cell membranes, in turn allowing more bacteria to interact with the CF. The same effect (partial detachment of CMCNa from SWCNTs) might also cause nonselective adsorption of bacteria on SWCNT surfaces by hydrophobic interactions, as seen for CNT-protein systems.^42^ Finally, in the current study we did not address the possible effects of extracellular polymeric substances (EPS) on EET. Electrically insulating EPS may be surrounding biofilms on the electrode, thus decreasing the efficiency of EET.

### 3.5 Electrochemical Impedance Spectroscopy Confirms the Findings from CAs

To find stronger evidence of EET exchange currents at the WE, we employed electrochemical impedance spectroscopy (EIS) on both abiotic and biotic samples. EIS has often been used to assess capacitive changes of electrodes after modifications with conducting materials, visible in the high-frequency range, and less commonly to detect EET exchange currents, typically visible at lower frequencies. The latter signature manifests itself in the Nyquist plot as the development of a second semicircle (a bending toward the real impedance axis), equivalent to a phase shift in the Bode plot toward 0°.^17,43^ A semicircle in Nyquist plots is, in general, indicative of an RC circuit behavior, i.e., a resistive and a capacitive contribution in parallel. The semicircle representing EET is much larger in size compared to the initial small semicircle measured at high frequencies (Figure S9), the latter typical for the electric double layer at electrode/electrolyte interfaces. To keep the EET process undisturbed, all EIS scans were performed immediately before or after a CA run while applying the same (DC) voltage bias of +0.2 V_Ag/AgCl_.

At medium to high frequencies of 20 Hz–20 kHz, Nyquist plots show an enlarged semicircle for “MR-1 SWCNT-HEQ”, while nearly no change or no significant change for the other SWCNTs compared to the abiotic “Control” (Figure S9). Usually, a larger semicircle would be indicative of a larger capacitive component of impedance, related to an increased EASA. However, based on large variations in the size of this small semicircle even for bare CF between different samples, we assume that in these experiments, the small semicircle is rather dominated by the experimental circumstances, such as the physical connection between the pores of CF and the Ti rod pierced through the CF, which is difficult to control.

**Figure 5:**
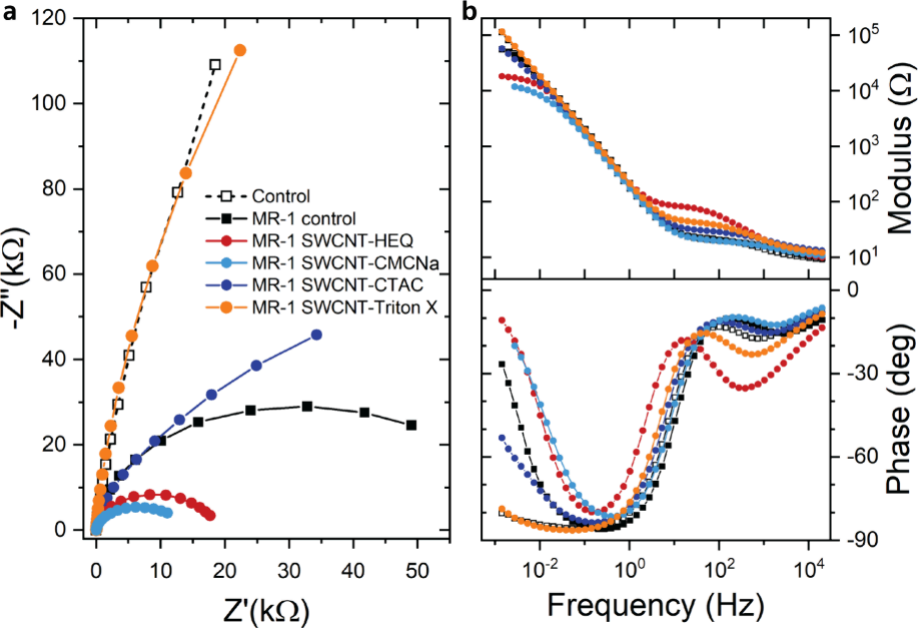
Electrochemical impedance spectrographs of bacterial reactors. (a) Nyquist and (b) Bode plots with (filled symbols) and without (empty symbols) *S. oneidensis* MR-1 bacteria (OD_600_ = 0.26) in the presence of SWCNTs functionalized by HEQ (red spheres), CMCNa (light blue spheres), CTAC (blue spheres), Triton X (orange spheres), and no SWCNTs present (control, black squares) in anoxygenic environment in M9 electrolyte with lactate (40 mM). Data were acquired immediately before or after CA shown in Figure 2. All EIS measurements were carried out at *V* = +0.2 V_Ag/AgCl_ to maintain bacterial EET. Note that for SWCNT-CMCNa, the two lowest frequencies were not recorded.

Next, we looked at the low-frequency component using Nyquist and Bode plots (Figure 5). In the abiotic MES, the low-frequency Nyquist plot points nearly straight upward, representing a typical diffusion-limited electrode/electrolyte interface (Figure 5a). The presence of EET clearly induced bending of the Nyquist plot toward the real impedance axis, corresponding to the upward shift of the phase angle toward 0° below 0.1 Hz on the Bode plot in all biotic samples, except for “MR-1 SWCNT-Triton X”. The latter was nearly identical to the abiotic control, in agreement with zero EET currents (Figure 2) and low OD_600_ values (Figure S5) of this sample (Figure 5b). Among the biotic samples, there was a marked difference in the degree of this change in impedance, i.e., a smaller and a larger second semicircle for “MR-1 SWCNT-HEQ” and “MR-1 SWCNT-CTAC” compared to “MR-1 control”, respectively, while we measured the smallest semicircle for “MR-1 SWCNT-CMCNa”. The smaller semicircle in this case indicates higher exchange currents at the WE, as can also be seen on the Bode plots where the largely overlapping “MR-1 SWCNT-CMCNa” and “MR-1 SWCNT-HEQ” are the closest among the variants in approaching a pure resistive character (i.e., closest to 0° phase) at frequencies below 10^–2^ Hz. To quantify the resistances of the WE to the exchange currents induced by bacteria (*R*_bac_) on selected samples, we fit the Bode plots shown in Figure 5b to an equivalent circuit model (Figure S1) previously used to characterize charge collectors coated by EET-bacteria.^44,45^ The values of *R*_bac_ were ∼65, ∼18.9, and ∼154 kΩ for “MR-1 control”, “MR-1 SWCNT-HEQ”, and “MR-1 SWCNT-CTAC”, respectively. The trend in these resistances clearly follows the CA and CV observations, indicating an improved electron transfer through the biofilm/electrode interface for the cellulose-derived functionalized SWCNTs. In other words, we expect that lowering *R*_bac_ will increase the electrical performance of potential MESs and MFCs.

## 4 Conclusions

Electrostatic attraction between bacteria and charge collectors is expected to increase the electrical performance of MESs and MFCs by recruiting more electroactive cells for performing EET. To realize this goal, we prepared positively charged SWCNTs to electrostatically attract the negatively charged cell membrane of *S. oneidensis* MR-1, and we used them as WEs in MESs. To validate this approach for functional groups of various sizes and charges, we prepared and characterized variants of SWCNTs wrapped by positively and negatively charged cellulose groups resulting in SWCNT-HEQ and SWCNT-CMCNa, respectively, and negatively charged and neutral smaller surfactants, resulting in SWCNT-CTAC and SWCNT-Triton X, respectively.

We found that all functional groups solubilized SWCNTs very well, preserving their structural integrity, which is essential for electrical and optical properties such as chirality, optical absorption, and emission, as verified by UV/vis/NIR absorption and 2D PL spectrometry. Importantly, HEQ, CMCNa, and CTAC induced Zeta potentials of +29, −65, and +52 mV on SWCNTs, comparable to that of the cell membrane of *S. oneidensis* MR-1 of −37.2 mV. To our surprise, MESs based on both SWCNT-HEQ and SWCNT-CMCNa, dissolved in the electrolyte outperformed those not containing SWCNTs in CA currents by 2.4-fold and 2.7-fold, respectively, after 90 h of operation with a continuously increasing trend. This effect also led to a significant decrease in resistance to exchange currents, as confirmed for SWCNT-HEQ by EIS. As we found by employing CV and EIS techniques as well as by microscopic observations of WEs, the enhanced performance with SWCNT-HEQ was due to both larger EASA of the WE (i.e., SWCNTs attaching to CF) and to preferential association of *S. oneidensis* MR-1 with SWCNT-HEQ. On the other hand, even though SWCNT-CMCNa did not attach to the CF, we observed a high number of bacteria on and around the CF when these SWCNTs were blended into MESs, in turn producing the highest current after ∼3 days of operation. In contrast, EET currents decreased or even diminished when small surfactant-based SWCNTs were mixed into the electrolyte compared to SWCNT-free MESs. The cause for this low performance seemed to originate in the aggregation of SWCNT-CTAC and the attachment of these SWCNTs to all electrode surfaces. While we did not observe such aggregation for SWCNT-Triton X, the low OD_600_ number in the electrolyte after CA and new redox peaks appearing in CV pointed toward a detrimental effect of Triton X on bacteria. In conclusion, although a clear understanding on how bacteria interact with charged cellulose-functionalized SWCNTs could not be established in the present work, our study shows that SWCNTs wrapped by both positively and negatively charged cellulose polymers can be interesting candidates for preparing bacterial bioelectrical interfaces for maximizing the harvested EET, with potential uses for low-scale energy harvesting and storage, sensing, and electrosynthesis devices.

## Supporting Information Available

Table of current levels extracted from cyclic voltammetry measurements in the cathodic regime; equivalent circuit model of the bioelectrode (Figure S1); selected Bode plots and impedance fits calculated according to the circuit model shown in Figure S1 (Figure S2); 2D PL mapping images of the solubilized SWCNTs with CMCNa, CTAC and Triton X (Figure S3); photographic snapshots of carbon felt electrodes with functionalized SWCNTs recorded before the addition of bacteria (Figure S4); optical density values of bulk electrolyte samples (Figure S5); individual CA curves of “MR-1 SWCNT-CMCNa” (Figure S6); photographic snapshots of carbon felt electrodes with and without chemically functionalized SWCNTs (Figure S7); combined bright field and fluorescence FITC microscopy images of carbon felt electrodes with bacteria with and without functionalized SWCNTs (Figure S8); Nyquist plots of bacterial reactors recorded at high-to-mid frequencies (Figure S9); and list of chiralities and corresponding wavelengths in Figure 1b,c (PDF)
