# Supplementary material for: Single-walled Carbon Nanotubes Wrapped with Charged Polysaccharides Enhance Extracellular Electron Transfer
Source: ACS Appl Bio Mater. 2024 Jul 30;7(8):5651–61. doi: 10.1021/acsabm.4c00749 (PMC11337164; doi:10.1021/acsabm.4c00749)
Supplement: Supplementary file 1 — mt4c00749_si_001.pdf [file mt4c00749_si_001.pdf]

Supporting Information

Single-walled carbon nanotubes wrapped with charged polysaccharides enhance extracellular electron transfer

Tomohiro Shiraki[1,2*], Yoshiaki Niidome[1], Arghyamalya Roy[3], Magnus Berggren[3,4], Daniel T. Simon[3], Eleni Stavrinidou[3,4], Gábor Méhes[3,5*]

1 Department of Applied Chemistry, Kyushu University, 744 Motooka, Nishi-ku, Fukuoka, 819-0395, Japan;

2 International Institute for Carbon-Neutral Energy Research (WPI-I2CNER), Kyushu University, 744 Motooka, Nishi-ku, Fukuoka, 819-0395, Japan;

3 Laboratory of Organic Electronics, Department of Science and Technology, Linköping University, 601 74 Norrköping, Sweden;

4 Wallenberg Wood Science Center, Department of Science and Technology, Linköping University, Norrköping, Sweden;

5 Graduate School of Information, Production and Systems, Waseda University, 2-7 Hibikino, Wakamatsu, Kitakyushu, Fukuoka 808-0135, Japan.

Corresponding Authors
* Gábor Méhes (mehes.gabor@aoni.waseda.jp); Tomohiro Shiraki (shiraki.tomohiro.992@m.kyushu-u.ac.jp)

Contents

1. Supplementary Table S1
2. Supplementary Figures S1–S9
3. List of chiralities and corresponding wavelengths in Figure 1b,c

1. Supplementary Table S1

Table S1. Current levels extracted from cyclic voltammetry measurements.

Funct. agent of SWCNTs	Abiotic @0.3V (μA)	Abiotic @0.3V {SWCNT/no-SWCNT}	Abiotic @0.2V (μA)	Biotic @0.2V (μA)	{Biotic/Abiotic} @0.2V	{Biotic -Abiotic} @0.2V (μA)
**no SWCNTs**	21.4	1.0	20.9	28.6	1.37	7.7
**HEQ**	48.8	2.28	48.3	65.4	1.35	17.1
**CMCNa**	22.4	1.05	25.0	59.0	2.36	34
**CTAC**	28.6	1.34	26.7	26.0	0.97	-0.7
**Triton-X**	17.7	0.83	15.6	24.2	1.55	8.6

2. Supplementary Figures S1–S9

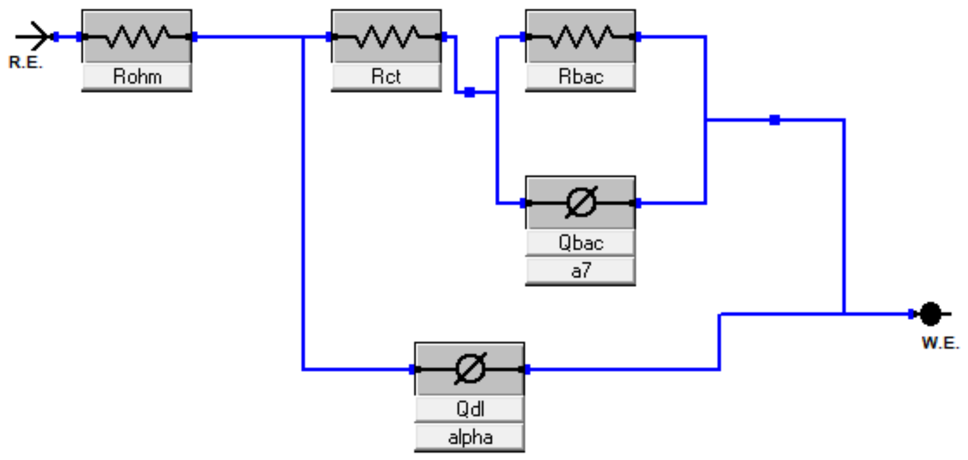

Figure S1. Equivalent circuit used to model the EIS data. R.E. and W.E. are reference and working electrodes, respectively. Rohm, Rct and Rbac are the ohmic, charge transfer and bacterial resistances, respectively. Qdl and Qbac are the constant phase elements representing the electric double layer and bacteria, respectively. a7 and alpha are constants describing the corresponding non-ideal capacitances.

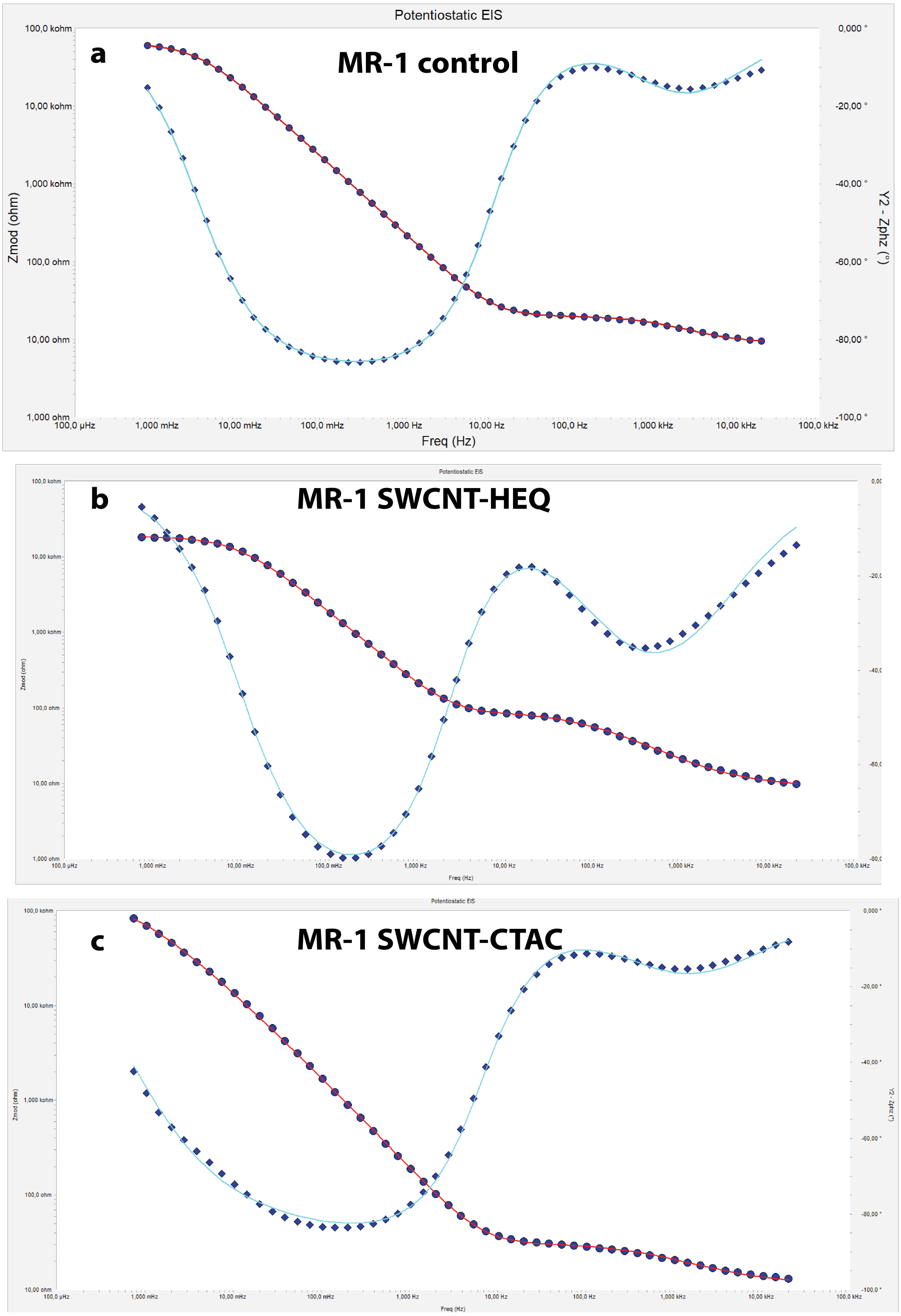

Figure S2. Selected Bode plots (symbols) shown also in Figure 5b and impedance fits (lines) calculated according to the circuit model shown in Figure S1 for a) MR-1 control, b) MR-1 SWCNT-HEQ and c) MR-1 SWCNT-CTAC. Spheres and red lines refer to modulus (left Y axis), while diamonds and blue lines to phase angle (right Y axis).

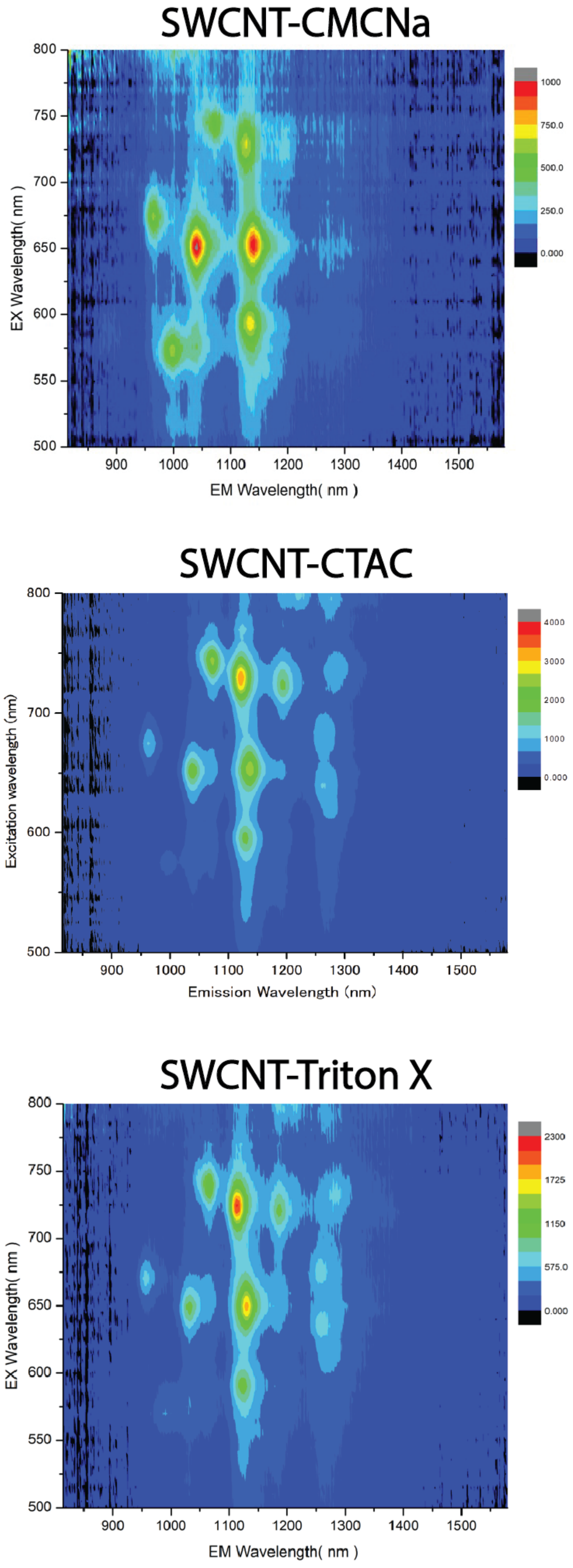

Figure S3. 2D PL mapping images of the solubilized SWCNTs with CMCNa, CTAC and Triton X.

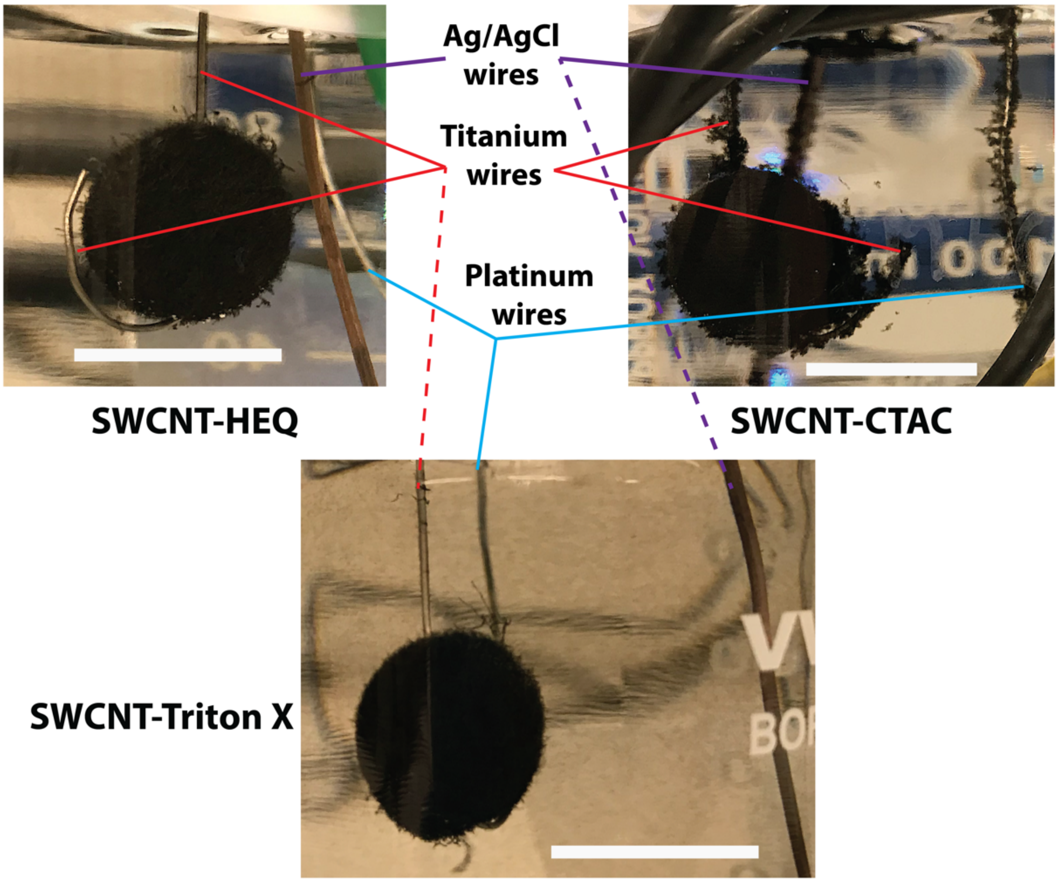

Figure S4. Photographic snapshots of carbon felt electrodes with functionalized SWCNTs recorded before the addition of bacteria. Scale bars are 1.2 cm.

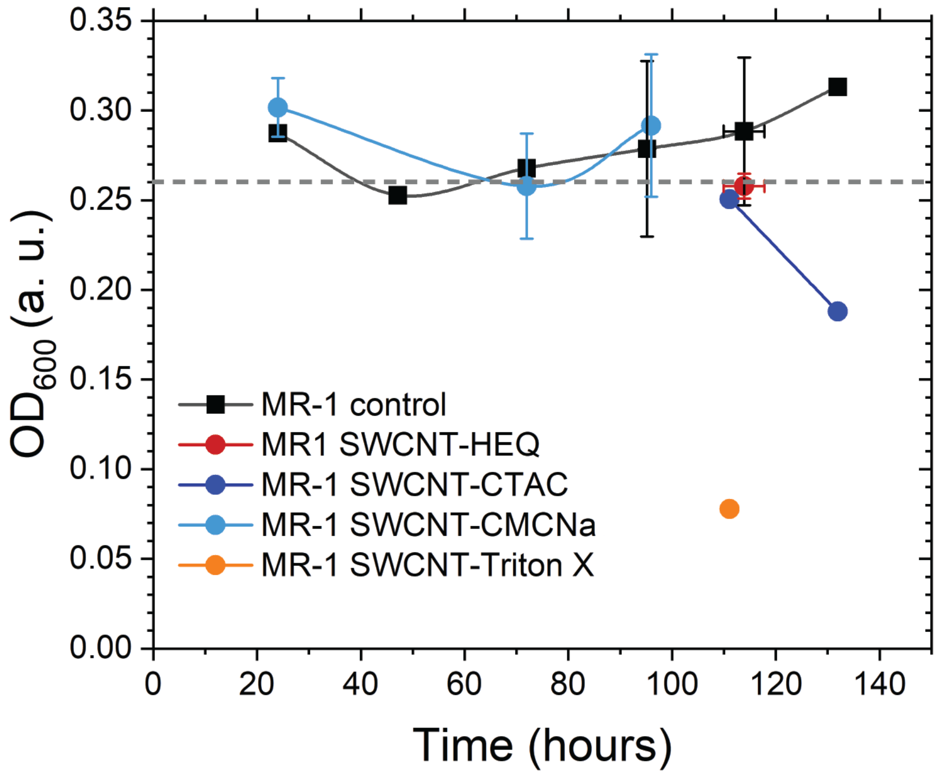

Figure S5. Background-corrected optical density values of bulk electrolyte samples obtained at 600 nm, sampled at various instances during chronoamperometric measurements at +0.2 $V_{Ag/AgCl}$ or immediately after the end of electrochemical experiments. For MR-1 control each of data points 4 and 5 in increasing order represents mean and standard deviation from $n = 2$ samples; for MR-1 SWCNT-HEQ the data point represents mean and standard deviation from $n = 2$ samples; for MR-1 SWCNT-CMCNa each data point represents mean and standard deviation from $n = 3$ samples; the rest of data points represent only one measured value. Dashed gray line denotes the value that was set as the initial OD_{600}.

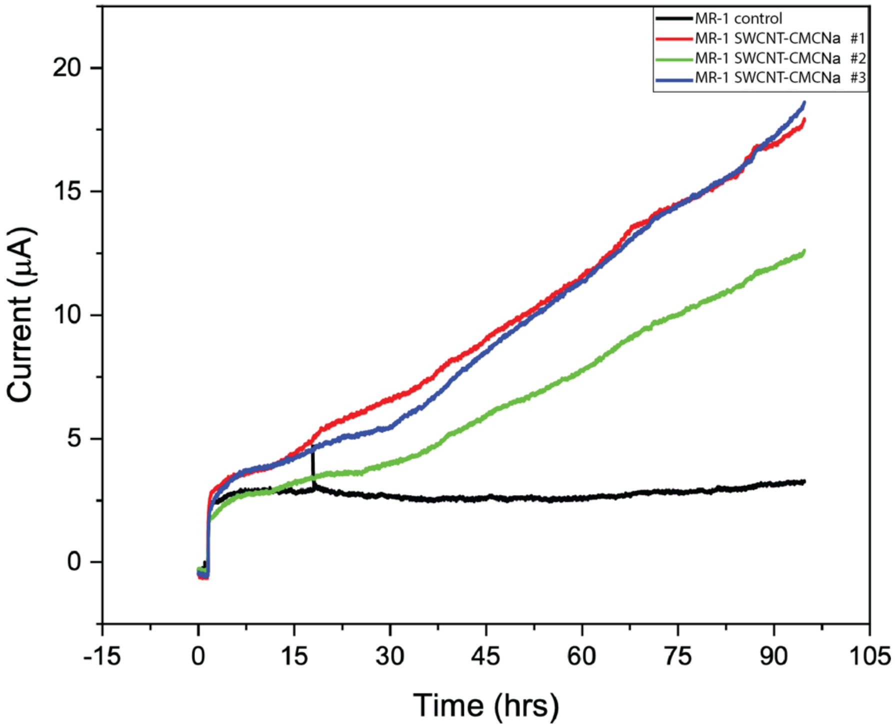

Figure S6. Chronoamperometric currents of the same batch of *S. oneidensis* MR-1 bacteria (OD_{600} = 0.26) in the presence of SWCNTs functionalized by CMCNa and no SWCNTs present, in anoxygenic environment in M9 electrolyte with lactate (40 mM) at +0.2 $V_{Ag/AgCl}$.

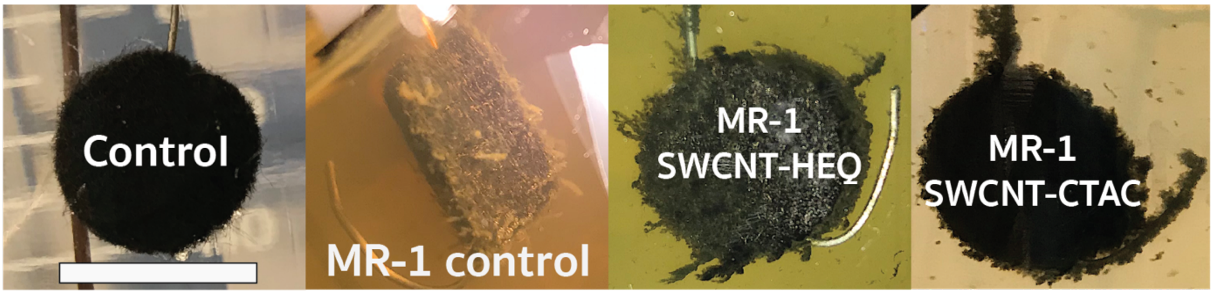

Figure S7. Visual observations of WEs. Photographic snapshots (WT bacteria, OD_{600} = 0.26) of carbon felt electrodes with and without chemically-functionalized SWCNTs. Scale bar is 1.2 cm. 'MR-1 control', 'MR-1 SWCNT-HEQ' and 'MR-1 SWCNT-CTAC' were taken ~110, ~117 and ~62 hours after the start of chronoamperometries, respectively.

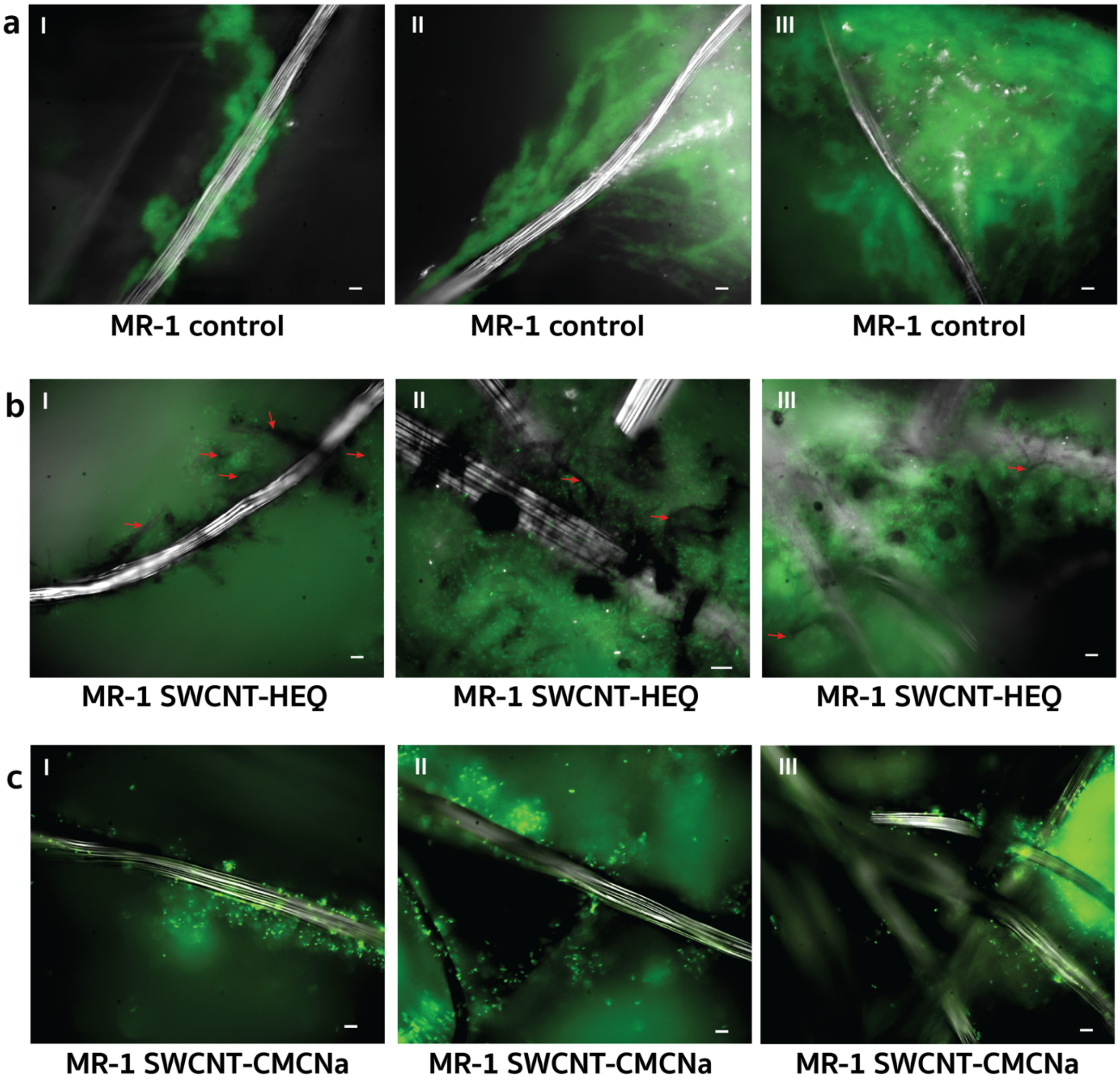

Figure S8. Combined bright field and fluorescence FITC microscopy images of carbon felt electrodes with and without functionalized SWCNTs. Green color marks the GFP mutant, wide silver rod-like structures are CF and thin black thread-like structures and areas (some of them marked by red arrows) in *b* are assumed to be aggregates of SWCNTs. Scale bars are 10 µm. a) I–III 'MR-1 control', b) I–III 'MR-1 SWCNT-HEQ' and c) I–III 'MR-1 SWCNT-CMCNa' images were taken after ~91, ~80, and ~100 hours of chronoamperometry, respectively.

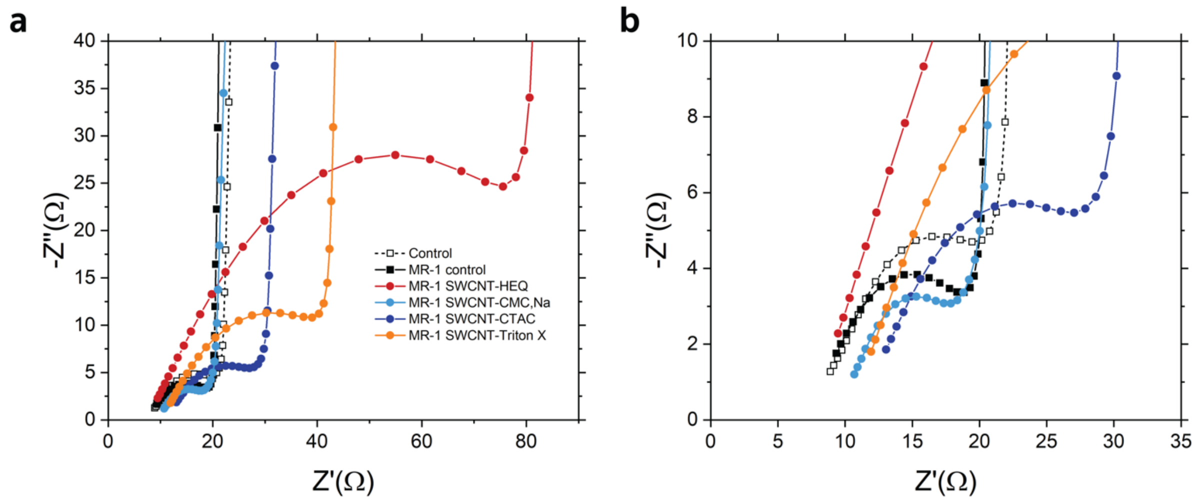

Figure S9. Nyquist plots of bacterial reactors with (filled symbols) and without (empty symbols) *S. oneidensis* MR-1 bacteria (OD_{600} = 0.26) in the presence of SWCNTs functionalized by HEQ (red spheres), CMCNa (light blue spheres), CTAC (blue spheres), Triton X (orange spheres) and no SWCNTs present (control; black squares) in anoxygenic environment in the electrolyte M9 with lactate (40 mM). *a* and *b* represent different magnifications of the same data. Data were acquired immediately before or after the CAs shown in Figure 2. All EIS measurements were carried out at +0.2 $V_{Ag/AgCl}$ to maintain bacterial EET.

2. List of chiralities and corresponding wavelengths in Figure 1b,c

In UV/vis/NIR absorption spectra (Figure 1b): For SWCNTs solubilized by HEQ, chiralities were observed at 971 nm for (8,3) chirality, 998 nm for (6,5) chirality, 1041 nm for (7,5) chirality, 1070 nm for (10,2) chirality, 1137 nm for (7,6) chirality, 1193 nm for (8,6) chirality and 1278 nm for (9,5) chirality. For other solubilizers, nearly identical absorption peaks were observed, showing that the solubilized SWCNTs were very similar to each other. This is because the used solubilizers have no chirality selectivity.

In 2D PL maps (Figure 1c): The observed spots were assigned to PL from SWCNT-HEQ with chiralities of (6,5) for λ_{ex} = 575 nm, λ_{em} = 999 nm, (8,4) for λ_{ex} = 595 nm, λ_{em} = 1136 nm, (10,3) for λ_{ex} = 640 nm, λ_{em} = 1273 nm, (7,5) for λ_{ex} = 655 nm, λ_{em} = 1041 nm, (7,6) for λ_{ex} = 655 nm, λ_{em} = 1143 nm, (8,3) for λ_{ex} = 675 nm, λ_{em} = 967 nm, (9,5) for λ_{ex} = 680 nm, λ_{em} = 1275 nm, (9,4) for λ_{ex} = 730 nm, λ_{em} = 1126 nm, (8,6) for λ_{ex} = 730 nm, λ_{em} = 1198 nm, (8,7) for λ_{ex} = 740 nm, λ_{em} = 1293 nm and (10,2) for λ_{ex} = 745 nm, λ_{em} = 1075 nm. For other solubilizers, similar PL signals were detected, showing that the solubilized SWCNTs were similar.
